# The complete chloroplast genome of *Holarrhena pubescens* and its phylogenetic analysis

**DOI:** 10.1080/23802359.2022.2162349

**Published:** 2023-02-16

**Authors:** Rushuang Xiang, Sijia Wang, Huihua Wan

**Affiliations:** a Key Laboratory of Beijing for Identification and Safety Evaluation of Chinese Medicine, Institute of Chinese Materia Medica, China Academy of Chinese Medical Sciences, Beijing, China; b College of Pharmaceutical, Dali University, Dali, China; c College of Pharmacy, Heilongjiang University of Chinese Medicine, Harbin, China; d School of Landscape Architecture, Beijing Key Laboratory of Ornamental Plants Germplasm Innovation & Molecular Breeding, National Engineering Research Center for Floriculture, Beijing Laboratory of Urban and Rural Ecological Environment, Engineering Research Center of Landscape Environment of Ministry of Education, Key Laboratory of Genetics and Breeding in Forest Trees and Ornamental Plants of Ministry of Education, Beijing Forestry University, Beijing, China

**Keywords:** *Holarrhena pubescens* Wall. ex G. Don, complete chloroplast genome, phylogenetic

## Abstract

*Holarrhena pubescens* Wall. ex G. Don, 1837 is an important medicinal plant belonging to the *Holarrhena* genus in the Apocynaceae family. In this study, the complete chloroplast (cp) genome sequence of *H. pubescens* was sequenced using the Illumina NovaSeq platform. The cp genome of *H. pubescens* was 160,108 bp in length with 37.21% overall GC content. The cp genome of *H. pubescens* containing a large single-copy region (LSC, 88,685 bp), a small single-copy region (SSC, 18,671 bp), and a pair of inverted repeat regions (SSC, 26,376 bp). The cp genome encoded 129 genes, including 84 protein-coding genes, 37 tRNA genes, and eight rRNA genes. Phylogenetic analysis based on complete protein coding genes sequences revealed that *H. pubescens* was closest to *Beaumontia murtonii*.

## Introduction

*Holarrhena pubescens* Wall. ex G.Don, 1837 is an important medicinal plant belonging to the *Holarrhena* genus in the Apocynaceae family (Khan et al. 2021; Namasudra et al. 2021), native to central and southern Africa, the Indian subcontinent, Indochina, and parts of China (Gupta et al. 2021). The bark of *H. pubescens* has been widely used to treat malaria, cough, cold, bronchopneumonia, dyspepsia, and diarrhea (Sharma et al. 2015; Gupta et al. 2021; Khan et al. 2021; Namasudra et al. 2021). *H. pubescens* is considered the most valuable medicine due to its numerous medicinal properties and nontoxic side effects (Zahara et al. 2020; Bala et al. 2022). Previous studies have focused on its pharmacological activity and chemical composition (Khan et al. 2021; Sripahco et al. 2021; Chowdhury and Mazumdar 2022). However, there is no report on the genomic studies of *H. pubescens*. Therefore, we present the complete chloroplast (cp) genome of *H. pubescens* for the first time, which will provide a theoretical and data foundation for the effective development, protection, and utilization of the *H. pubescens*.

## Materials and methods

### Plant material and DNA sequencing

**Figure 1.**
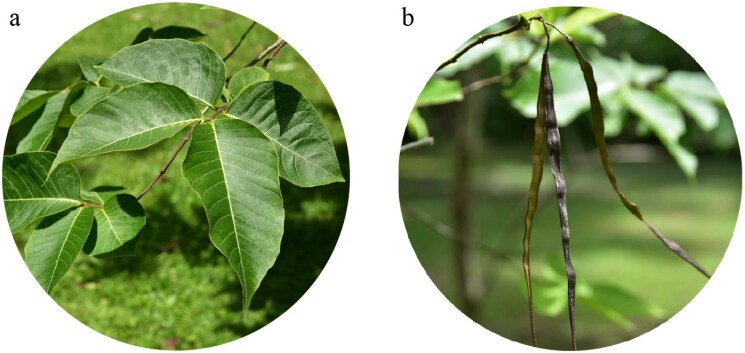
(a) The leaves of *H. pubescens*. (b) The follicles of *H. pubescens*. The leaf blade of this species is ovate or elliptic, with 10–15 pairs of lateral veins, and the follicles of this species are linear, with white, punctate lenticels. The photos of *H. pubescens* were taken by the authors in Xishuangbanna county, Yunnan Province, China.

This article is licensed under the Yunnan Province Biodiversity Conservation Regulations and with permission from Xishuangbanna (Yunnan Province, China), and the Institute of Chinese Materia Medica, China Academy of Chinese Medical Sciences (Beijing, China). Fresh leaves of *H. pubescens* were collected from Xishuangbanna City (N21°55′40″, E101°15′12″), Yunnan Province, China (Figure 1). We have obtained permission from the local forestry department to collect samples. This study was conducted in accordance with the laws of the People’s Republic of China. This specimen was identified by Kunming Cai Zhi Biotechnology Co. (Kunming, China). The voucher specimen was deposited in the herbarium of the Institute of Chinese Materia Medica, China Academy of Chinese Medical Sciences (Xiangxiao Meng, xxmeng@icmm.ac.cn) under the voucher number: XSBN20220524. Genomic DNA was extracted using the Plant Genomic DNA Kit (Tiangen, Beijing, China). After the sample is tested as qualified, take 0.5 μg for each sample DNA template, according to the manufacturer’s instructions (Illumina NovaSeq 6000), construct a short fragment insertion library (insertion size 350–500 bp) for sequencing, run the paired-end sequencing program (PE), and obtain 150 bp paired-end sequencing reads.

### Genome assembly and annotation

The high-quality reads were assembled with GetOrganelle v.1.7.5 (Jin et al. 2020) and annotated by CPGAVAS2 (Shi et al. 2019). The annotated cp genome was deposited in the Genome Warehouse (accession number: GWHBKKE01000000).

### Phylogenetic analysis

To evaluate the phylogenetic position of *H. pubescens*, 11 complete cp genome sequences from Apocynaceae and one outgroup species (*Gentiana crassicaulis*) were downloaded from the NCBI database. The complete protein coding genes of 13 species were extracted and aligned by MAFFT v7.471 (Katoh and Standley 2013). Maximum-likelihood (ML) analyses were performed with RAxML v8.2.12 (Stamatakis 2014) using the rapid bootstrap algorithm with 1000 replicates to assess branch support, combined with a search of the best-scoring ML tree under default parameters.

## Results and discussion

### Genome structure analysis

**Figure 2.**
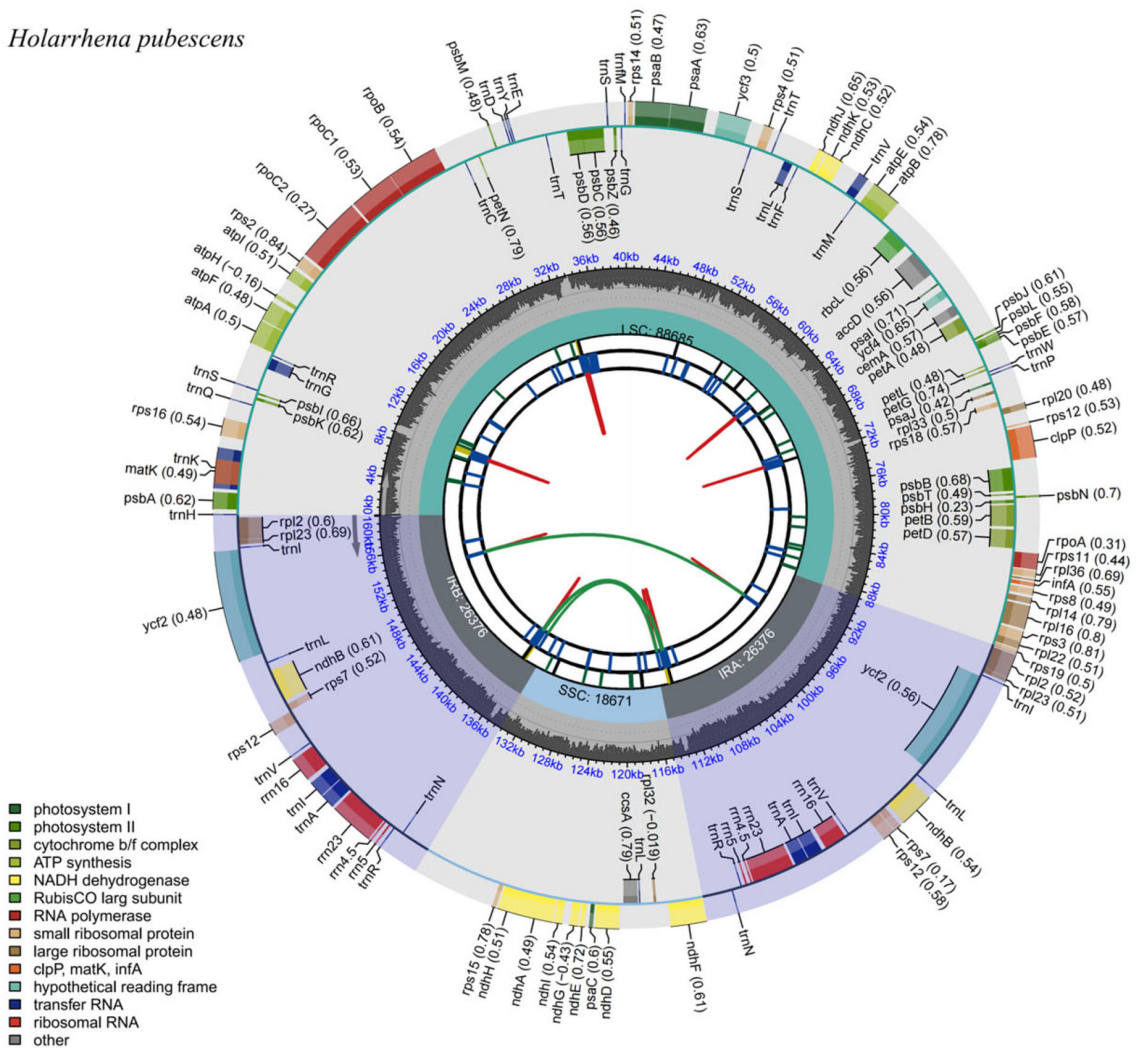
Chloroplast reference genome of *Holarrhena pubescens*. This figure has six circles from the center to the outside. The first circle shows the forward and reverse repeats connected with the red and green arcs, respectively. The second circle and the third circle show tandem repeats and microsatellite sequences marked with short strips, respectively. The fourth circle indicates the position of LSC, SSC, IRA, and IRB, and the fifth circle indicates the GC content. The genes outside the sixth circle are transcribed counterclockwise, while the genes inside are transcribed clockwise. Genes belonging to different functional groups are color coded, as shown in the left corner.

In total, 10.4 Gb of raw data were obtained (69,706,856 reads). The sequences obtained from sequencing are filtered with low quality data to obtain clean reads (67,006,166 reads), and based on the clean reads, an appropriate amount of data is used for assembly and subsequent analysis to finally obtain the complete genome sequence, the genome assembly coverage is 100% (Figure S1). The complete cp genome of *H. pubescens* is 160,108 bp in length (Figure 2) and includes a pair of inverted repeats 26,376 bp long, separated by a small and a large single-copy region of 18,671 bp and 88,685 bp, respectively. A total of 129 genes with 84 coding sequences, 37 tRNA genes, and eight unique rRNA sequences were identified. Among them, the *ndhD* gene is an RNA editing gene, using ACG as the start codon, which was predicted based on the start codon and the length of the gene of the *ndhD* gene of the closely related species *Nerium oleander* L. (NC_025656). The GC content of the cp genome was 37.21%.

### Phylogenetic analysis

**Figure 3.**
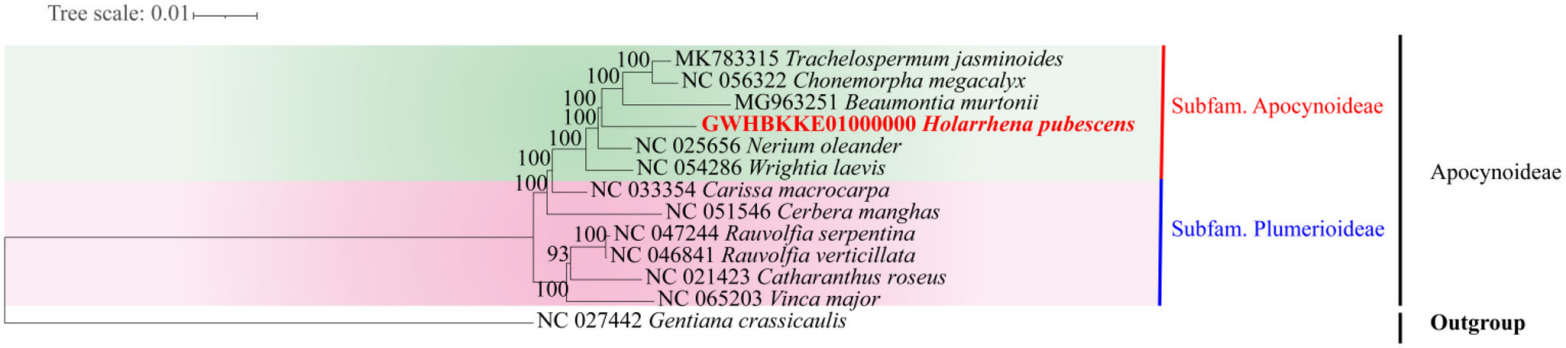
Phylogenetic analysis of 12 species and one taxon as outgroups based on complete protein coding genes sequences by RAxML, bootstrap support value near the branch. The following sequences were used: MK783315 *Trachelospermum jasminoides* (Wang et al. 2019), NC_056322 *Chonemorpha megacalyx* (Deng et al. 2021), MG963251 *Beaumontia murtonii* (Fishbein et al. 2018), GWHBKKE01000000 *Holarrhena pubescens,* NC_025656 *Nerium oleander* (Straub et al. 2014), NC_054286 *Wrightia laevis* (Li et al. 2020), NC_033354 *Carissa macrocarpa* (Jo et al. 2017), NC_051546 *Cerbera manghas* (Liao et al. 2020), NC_047244 *Rauvolfia serpentina* (Zhang et al. 2021), NC_046841 *Rauvolfia verticillata* (Chen et al. 2019), NC_021423 *Catharanthus roseus* (Ku et al. 2013), NC_065203 *Vinca major* (Hendrian 2007), and NC_027442 *Gentiana crassicaulis* (Ni et al. 2017).

Phylogenetic analysis revealed that *H. pubescens* was closely related to *Beaumontia murtonii* Craib (Figure 3). The cp genome sequence of *H. pubescens* in this study might provide important information for phylogenetic and evolutionary studies in Apocynaceae.

## Supplementary Material

Supplemental Material

## Data Availability

The genome sequence data that support the findings of this study are openly available in the Genome Warehouse of NGDC at https://ngdc.cncb.ac.cn/gwh under the accession number: GWHBKKE01000000. The associated BioProject, GSA, and Bio-Sample numbers are PRJCA011728, CRA008103, and SAMC873599, respectively.

## References

[CIT0001] Bala K, Melkani I, Singh AP, Singh AP, Kaur J. 2022. *Holarrhena antidysenterica* in inflammatory bowel disease: a potential review. J Drug Deliv Ther. 12(4):221–226.

[CIT0002] Chen W, Liang W, Li A, Ma J. 2019. Characterization of the complete plastid genome of *Rauvolfia verticillata* (Apocynaceae), with its phylogenetic analysis. Mitochondrial DNA B Resour. 4(2):4190–4191. 33366377 10.1080/23802359.2019.1693287 PMC7707761

[CIT0003] Chowdhury SK, Mazumdar T. 2022. The pesticidal activities of *Rhizospheric Bacteria* isolated from *Holarrhena pubescens*, their plant growth promotion and IAA production optimization. Sci J Biol. 1(5):10–21.

[CIT0004] Deng YP, Zhang XL, Li LY, Yang T, Liu GH, Fu YT. 2021. Characterization of the complete mitochondrial genome of the swine kidney worm *Stephanurus dentatus* (Nematoda: Syngamidae) and phylogenetic implications. Vet Parasitol. 295:109475. 34062343 10.1016/j.vetpar.2021.109475

[CIT0005] Fishbein M, Livshultz T, Straub S, Simoes AO, Boutte J, McDonnell A, Foote A. 2018. Evolution on the backbone: Apocynaceae phylogenomics and new perspectives on growth forms, flowers, and fruits. Am J Bot. 105(3):495–513. 29733432 10.1002/ajb2.1067

[CIT0006] Gupta N, Choudhary SK, Bhagat N, Karthikeyan M, Chaturvedi A. 2021. In silico prediction, molecular docking and dynamics studies of steroidal alkaloids of *Holarrhena pubescens* Wall. ex G. Don to guanylyl cyclase C: implications in designing of novel antidiarrheal therapeutic strategies. Molecules. 26(14):4147. 34299422 10.3390/molecules26144147 PMC8305770

[CIT0007] Hendrian KK. 2007. Molecular phylogeny of *Ochrosia sensu* lato (Apocynaceae) based on rps16 intron and ITS sequence data: supporting the inclusion of *Neisosperma*. Chromosome Bot. 4(2):133–140.

[CIT0008] Jin JJ, Yu WB, Yang JB, Song Y, DePamphilis CW, Yi TS, Li DZ. 2020. GetOrganelle: a fast and versatile toolkit for accurate de novo assembly of organelle genomes. Genome Biol. 21(1):241. 32912315 10.1186/s13059-020-02154-5 PMC7488116

[CIT0009] Jo S, Kim HW, Kim YK, Cheon SH, Kim KJ. 2017. The complete plastome sequence of *Carissa macrocarpa* (Eckl.) A. DC. (Apocynaceae). Mitochondrial DNA B Resour. 2(1):26–28. 33473704 10.1080/23802359.2016.1233468 PMC7800814

[CIT0010] Katoh K, Standley DM. 2013. MAFFT multiple sequence alignment software version 7: improvements in performance and usability. Mol Biol Evol. 30(4):772–780. 23329690 10.1093/molbev/mst010 PMC3603318

[CIT0011] Khan S, Viquar U, Alam MA, Moin MS, Khatoon F, Minhajuddin A. 2021. Ethno-pharmacology of *Holarrhena antidysenterica* wall. ex g. don. (Tewāj) in light of unani system of medicine. Int J Bot Stud. 5(6):1133–1139.

[CIT0012] Ku C, Chung WC, Chen LL, Kuo CH. 2013. The complete plastid genome sequence of madagascar periwinkle *Catharanthus roseus* (L.) G. Don: plastid genome evolution, molecular marker identification, and phylogenetic implications in asterids. PLoS One. 8(6):e68518. 23825699 10.1371/journal.pone.0068518 PMC3688999

[CIT0013] Li LM, Fu JX, Song XQ. 2020. Complete plastome sequence of *Wrightia laevis* Hook. f. a dyestuff species. Mitochondrial DNA B Resour. 3(5):2533–2534. 10.1080/23802359.2020.1778578 PMC7782915 33457852

[CIT0014] Liao M, Wei XF, Ding HP, Tang GD. 2020. The complete chloroplast genome of the highly poisonous plant *Cerbera manghas* L. (Apocynaceae). Mitochondrial DNA B Resour. 5(3):3084–3085. 33553627 10.1080/23802359.2020.1794994 PMC7850440

[CIT0015] Namasudra S, Phukan P, Bawari M. 2021. GC–MS analysis of bioactive compounds and safety assessment of the ethanol extract of the barks of *Holarrhena pubescens* Wall. ex.G.Don (family Apocynaceae): sub-acute toxicity studies in swiss albino mice. Pharmacogn J. 13(1):162–171.

[CIT0016] Ni L, Zhao Z, Xu H, Chen S, Dorje G. 2017. Chloroplast genome structures in Gentiana (Gentianaceae), based on three medicinal alpine plants used in Tibetan herbal medicine. Curr Genet. 63(2):241–252. 27422574 10.1007/s00294-016-0631-1

[CIT0017] Sharma DK, Gupta VK, Kumar S, Joshi V, Mandal RS, Prakash AG, Singh M. 2015. Evaluation of antidiarrheal activity of ethanolic extract of *Holarrhena antidysenterica* seeds in rats. Vet World. 8(12):1392–1395. 27047049 10.14202/vetworld.2015.1392-1395 PMC4774815

[CIT0018] Shi L, Chen H, Jiang M, Wang L, Wu X, Huang L, Liu C. 2019. CPGAVAS2, an integrated plastome sequence annotator and analyzer. Nucleic Acids Res. 47(W1):W65–W73. 31066451 10.1093/nar/gkz345 PMC6602467

[CIT0019] Sripahco T, Tovaranonte J, Pripdeevech P. 2021. Chemical composition and antimicrobial activity of essential oil of *Holarrhena pubescens* flowers. Chem Nat Compd. 57(4):781–783.

[CIT0020] Stamatakis A. 2014. RAxML version 8: a tool for phylogenetic analysis and post-analysis of large phylogenies. Bioinformatics. 30(9):1312–1313. 24451623 10.1093/bioinformatics/btu033 PMC3998144

[CIT0021] Straub SC, Moore MJ, Soltis PS, Soltis DE, Liston A, Livshultz T. 2014. Phylogenetic signal detection from an ancient rapid radiation: effects of noise reduction, long-branch attraction, and model selection in crown clade Apocynaceae. Mol Phylogenet Evol. 80:169–185. 25109653 10.1016/j.ympev.2014.07.020

[CIT0022] Wang H, Cheng X, Chen W, Li L, Chen L. 2019. Complete plastome sequence of *Trachelospermum jasminoides* (Lindley) Lemaire (Apocynaceae). Mitochondrial DNA B Resour. 1(4):2086–2087.

[CIT0023] Zahara K, Panda SK, Swain SS, Luyten W. 2020. Metabolic diversity and therapeutic potential of *Holarrhena pubescens*: an important ethnomedicinal plant. Biomolecules. 10(9):1341. 32962166 10.3390/biom10091341 PMC7565871

[CIT0024] Zhang K, Liu L, Shan X. 2021. Characterization of the complete chloroplast genome of *Tabernaemontana divaricata* (Apocynaceae), a valuable and endangered plant. Mitochondrial DNA B Resour. 6(11):3125–3126. 34746383 10.1080/23802359.2021.1984331 PMC8567903

