# Supplementary material for: The complete chloroplast genome of Holarrhena pubescens and its phylogenetic analysis
Source: Mitochondrial DNA B Resour. 2023 Feb 16;8(2):266–9. doi: 10.1080/23802359.2022.2162349 (PMC9937002; doi:10.1080/23802359.2022.2162349)
Supplement: Supplemental Material [file TMDN_A_2162349_SM7851.docx]

**Figure S1 The assembly coverage plot for the *Holarrhena pubescens* complete chloroplast genome.**

**
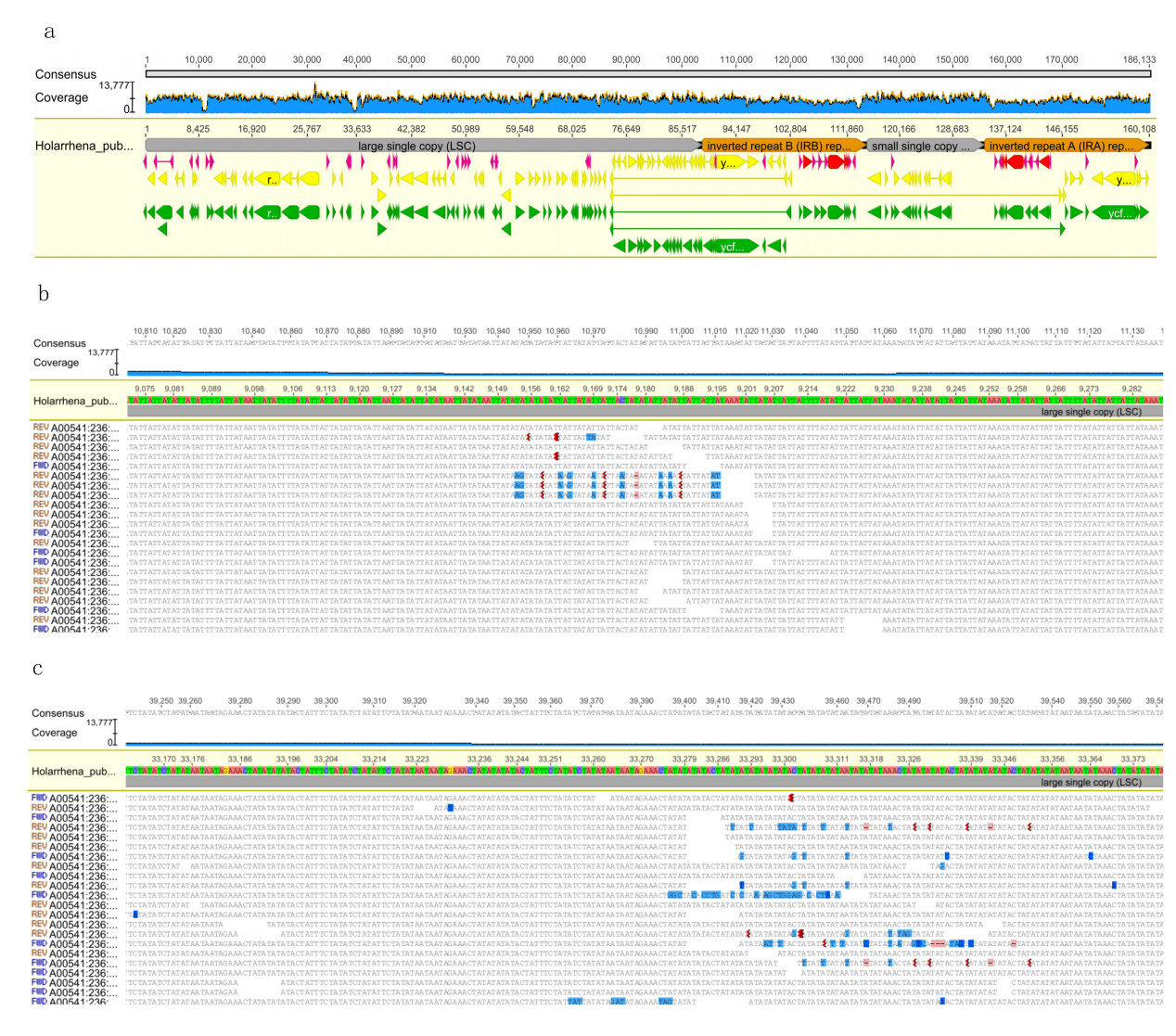
**

Figure S1 The assembly coverage plot for the *Holarrhena pubescens* complete chloroplast genome. This graph was generated using Genious R9（V9.0.2） by mapping sequenced clean reads to the assembled chloroplast genome (a) Alignment when sequencingclean reads mapping into the complete chloroplast genome of assembled *Holarrhena pubescens.* (b) Alignment in the *H. pubescens* chloroplast genome at 9180 bp when sequencingclean reads mapping into the assembled chloroplast genome. (c) Alignment in the *H. pubescens* chloroplast genome at 33286 bp when sequencingclean reads mapping into the assembled chloroplast genome. A picture is divided into upper and lower parts. The upper part is blue, representing the assembly coverage, and the lower part is the genome annotation result. The length and position of the gray arrow represent the position of the large single-copy region and the small single-copy region. The length and position of the orange arrows represent the position of a pair of inverted repeat regions. Genes are represented by green arrows, CDS is encoded by yellow arrows, rRNA is encoded by red arrows, and tRNA is encoded by pink arrows.
